# *Clostridioides difficile* Colonization and Infection in Pediatric Oncology and Stem Cell Transplant Patients

**DOI:** 10.1093/ofid/ofag149

**Published:** 2026-03-21

**Authors:** Daniel N Willis, Erik R Dubberke, Robert J Hayashi, Phillip I Tarr, David B Haslam, Tiffany Hink, Jingqin Luo, Yu Tao, Amruta Padhye, Erin M Hall, Gregory A Storch

**Affiliations:** Department of Pediatrics, Washington University School of Medicine, St Louis, Missouri, USA; Department of Medicine, Washington University School of Medicine, St Louis, Missouri, USA; Department of Pediatrics, Washington University School of Medicine, St Louis, Missouri, USA; Department of Pediatrics, Washington University School of Medicine, St Louis, Missouri, USA; Department of Molecular Microbiology, Washington University School of Medicine, St Louis, Missouri, USA; Department of Pediatrics, College of Medicine, University of Cincinnati, Cincinnati, Ohio, USA; Department of Medicine, Washington University School of Medicine, St Louis, Missouri, USA; Department of Surgery, Washington University School of Medicine, St Louis, Missouri, USA; Department of Surgery, Washington University School of Medicine, St Louis, Missouri, USA; Department of Pediatrics, School of Medicine, University of Missouri, Columbia, Missouri, USA; Department of Pediatrics, Children's Mercy Hospital, Kansas City, Missouri, USA; Department of Pediatrics, Washington University School of Medicine, St Louis, Missouri, USA

## Abstract

**Background:**

Pediatric oncology and hematopoietic stem cell transplant (HSCT) patients have elevated risk for *Clostridioides difficile* infection (CDI), which can prolong hospitalization and delay chemotherapy. Colonization is an important prelude to symptomatic CDI. We sought to characterize colonization status in these patients.

**Methods:**

We retrospectively studied 276 stools longitudinally collected over 34 months from 32 HSCT and 12 oncology patients treated at a single tertiary center. Specimens were cultured for *C difficile* and compared by whole genome sequencing. The fecal microbiome was characterized by 16S rRNA gene sequencing.

**Results:**

Baseline cultures were positive in 16 (50%) HSCT patients and 2 (12%) oncology. On subsequent samples, 64% of patients who were initially negative acquired colonization: 8 of 15 (53%) HSCT and 8 of 10 (80%) oncology. Nine clonal strains and 25 multilocus sequence types were identified by whole genome sequencing, with 4 clones found in both cohorts. Nine patients had different strains at different time points. Seven clonal strains were found in multiple patients. Seven (15.9%) patients had symptomatic CDI. *C difficile*–positive stools had greater microbial diversity than negative stools in both the oncology cohort (Simpson diversity index, 0.07; 95% CI, .01–.14; *P* = .03) and the HSCT cohort (0.15; 95% CI, .07–.24; *P* < .001).

**Conclusions:**

*C difficile* acquisition and colonization are common in pediatric oncology and HSCT patients. The high prevalence of clonally related strains in multiple patients suggests that asymptomatic patients may be important reservoirs of this pathogen and lead to symptomatic CDI in some patients. Gut microbial composition may influence the risk of colonization.

## Teaser

*Clostridioides difficile* infection and colonization are common in pediatric oncology patients. We demonstrate that colonization status may be transient and episodic. This turnover may contribute to colonization spread and subsequent *C difficile* infection. Microbiome diversity was associated with *C difficile* colonization.

## BACKGROUND

Symptomatic *Clostridioides difficile* infection (CDI) is associated with significant morbidity and mortality in hospitalized patients [1]. CDI is characterized by *C difficile* toxin production and inflammation in the intestine resulting in a range of symptoms including colitis and diarrhea. In general, *C difficile* has less impact on children as compared with adults, and children often excrete *C difficile* without symptoms [2]. Additionally, detection of *C difficile* is common in young asymptomatic children, especially those <1 year of age [2]. This provides important context for interpreting *C difficile* test results. However, some pediatric patients are at higher risk for CDI and poor outcomes [1, 3]. Notably, CDI in pediatric oncology patients is associated with longer hospital stays and delayed chemotherapy [4, 5], potentially compromising cancer control.

*C difficile* colonization is a prelude to CDI and contributes to transmission [6]. *C difficile* colonization is defined as the presence of *C difficile* in the gut of an asymptomatic individual. Colonization rates rise with increasing hospital exposure [7]. In patients of any age who are immunocompromised, the rate of colonization may be several times greater than in healthy hosts [6, 8]. This observation has 2 important implications. First, colonized patients are at increased risk for subsequent symptomatic CDI [9]. Second, colonized patients can shed pathogenic *C difficile*, which can then colonize others and cause symptomatic infection in those recipients [10]. The progression from colonization to CDI is incompletely understood and requires further attention.

Microbial community composition is associated with CDI and *C difficile* colonization and may herald symptomatic infection. The microbiome is a complex “ecosystem” that regulates the host immune response. However, if disrupted, the gut microbiome can permit pathogens to proliferate [11–13]. Lee et al demonstrated an association between the presence of bacterial groups and CDI in adult hematopoietic stem cell transplant (HSCT) patients. While Bacteroidetes, Lachnospiraceae, and Ruminococcaceae were identified as protective, *Enterococcus faecalis* was associated with increased risk of CDI [14]. In this study, we sought to describe the prevalence of *C difficile* colonization in pediatric oncology and HSCT patients at a single tertiary children's hospital and to characterize the acquisition and loss of colonization. We additionally aimed to evaluate for common strains among patients. Finally, we described the relationship between gut microbiome diversity and *C difficile* colonization and explored any associated bacterial families.

## METHODS

This study utilized frozen samples from 2 previous research cohorts. As the present study used deidentified banked samples and data, it was designated as exempt research by the Washington University Human Research Protection Office. The first cohort consisted of children who underwent autologous or allogeneic HSCT for malignant or nonmalignant indications. Most in this cohort had previous admissions and/or chemotherapy. The primary aim of this study was to evaluate the stool microbiome in setting of HSCT, with samples obtained before transplant, throughout engraftment, and in immediate follow-up. Patients were enrolled from 2015 to 2017. The second cohort consisted of children with new oncologic diagnoses, each of whom provided a stool sample before initiating chemotherapy and longitudinally, with the primary aim of evaluating the stool microbiome during chemotherapy and with episodes of febrile neutropenia. Patients were enrolled from 2017 to 2018. Both cohorts were treated by the single pediatric oncology program at St Louis Children's Hospital, with shared clinical spaces, nursing staff, and adjacent inpatient units. Stools were collected from patients in inpatient or outpatient settings. The outpatient clinic and inpatient service are on the same floor of the hospital. All stools from the previous studies had been stored at −80 °C for up to 72 months. Databases were available that comprised demographic variables, including age, sex, and ethnicity, as well as clinical variables, including concurrent infections, underlying malignancy, and treatment regimen. Samples were available at varying intervals. No more than 1 sample per week was selected for determination of colonization status and microbiome composition.

### *C difficile* Testing

For each specimen, 1 g of frozen stool was chipped and heat shocked at 80 °C for 10 minutes. Samples were inoculated into cycloserine-cefoxitin mannitol with taurocholate, lysozyme, and cysteine broth (Anaerobe Systems), which were incubated under anaerobic conditions and monitored for growth. Positive broths were subcultured onto sheep blood agar plates, and broths that remained negative were subcultured on day 7 [15]. Colonies that were morphologically consistent with *C difficile* were confirmed by matrix-assisted laser desorption ionization–time of flight (BioMérieux), and isolates were frozen at −80 °C until further analysis.

### Strain Typing

Whole genome sequencing (WGS) was performed on all *C difficile* isolates [16]. Sequence libraries were created with Illumina Nextera XT reagents as described in the manufacturer's protocol. Samples were sequenced to a depth of at least 5 million paired-end 150-nucleotide reads per sample. WGS analysis was conducted to determine the genetic relatedness of the isolates. Initially, raw paired-end reads were subjected to quality control via Trimmomatic (version 0.39) to remove low-quality bases and adapter sequences. The quality of the trimmed reads was then verified with FastQC. De novo genome assembly was performed on the quality-filtered reads by the Unicycler assembler (version 0.5.0). The resulting assemblies were assessed for quality and contiguity by using QUAST against the *C difficile* 630 reference genome (GCF_000009205.2) and for completeness and contamination by using CheckM. For comparative analysis, the assembled genomes were typed per multilocus sequence typing (MLST) and functionally annotated with Prokka (version 1.14.6). A pangenome analysis was conducted in Panaroo (version 1.3.4) to identify the core and accessory gene content. To infer high-resolution phylogenetic relationships, a core single-nucleotide polymorphism (SNP) analysis was conducted. Trimmed reads were mapped to the reference genome to call SNPs via the Snippy pipeline (version 4.6.0). A core SNP alignment was generated by snippy-core, and a maximum likelihood phylogenetic tree was constructed from this alignment in FastTree (version 2.1.11). The resulting variant call format file was filtered with BCFtools, and a SNP distance matrix was generated for further downstream analysis and visualization in R. Identification of clonal strains based on SNP analysis supplied evidence of transmission networks and environmental contamination at a more granular level than what MLST provides, and its use has been demonstrated in prior studies [17]. Clonal strains were defined as isolates that differed by <2 core MLST SNPs, per prior studies. MLST was performed with the MLST software package [18].

A descriptive analysis of *C difficile* colonization was conducted with raw counts and percentages reported for initial colonization status, subsequent colonization, and a descriptive evaluation of interpersonal bacterial spread. “Initial” was defined as the first stool collected during the study period.

### Microbiome Analysis

Nucleic acid extraction was carried out via Qiagen PowerLyzer PowerSoil extraction kits (MO BIO Laboratories). 16Sv4 rRNA gene sequencing was performed on all specimens at the Alkek Center for Metagenomics and Microbiome Research, Department of Molecular Virology and Microbiology, Baylor College of Medicine, Houston, Texas. Microbiome diversity was characterized by Shannon and Simpson diversity indices with repeated measures linear mixed effects modeling applied to each summary measure (Shannon diversity, Simpson, inverse Simpson, and Chao indexes). Comparison was made at the specimen level by comparing *C difficile*–positive stools to negative stools, incorporating the fixed effect of positive colonization status and the random effect of patients to account for the repeated measurements from the same patient [19, 20]. The least square mean difference between colonized and uncolonized samples was reported with lower and upper 95% CIs and *P* values testing the estimated least square mean difference against 0. All *P* values were 2-tailed. Raw count data at the family level was compared between colonized and negative samples through DESeq2 negative binomial distribution analysis. Effect size was expressed in shrunken log fold change, and *P* values were adjusted to control for the false discovery rate [21].

## RESULTS

We analyzed 276 samples from 44 patients: 158 were from 32 HSCT patients, 20 of whom had underlying malignancies and 12 of whom had nonmalignant indications for transplant; 118 were from 12 newly diagnosed oncology patients. The demographic characteristics of each group are summarized in Table 1. Characteristics of colonized patients, stratified by timing of colonization, are summarized in Supplementary Table 1.

**Table 1.** Patient Characteristics of Pediatric HSCT and Oncology Patients

	No. (%) or Median (IQR)	
Cohort	HSCT	Oncology
Patients	32	12
Age at initial sample, y	7.5 (1.75–12.25)	3.5 (1.75–9.5)
Diagnosis		
Leukemia	8 (25)	7 (58.3)
Lymphoma	3 (9.4)	0 (0)
Solid tumor	9 (28.1)	5 (41.6)
Nonmalignant	12 (37.5)	…
*Clostridioides difficile* colonization at baseline	16 (50)	2 (16)

Abbreviation: HSCT, hematopoietic stem cell transplant.

**Figure 1.**
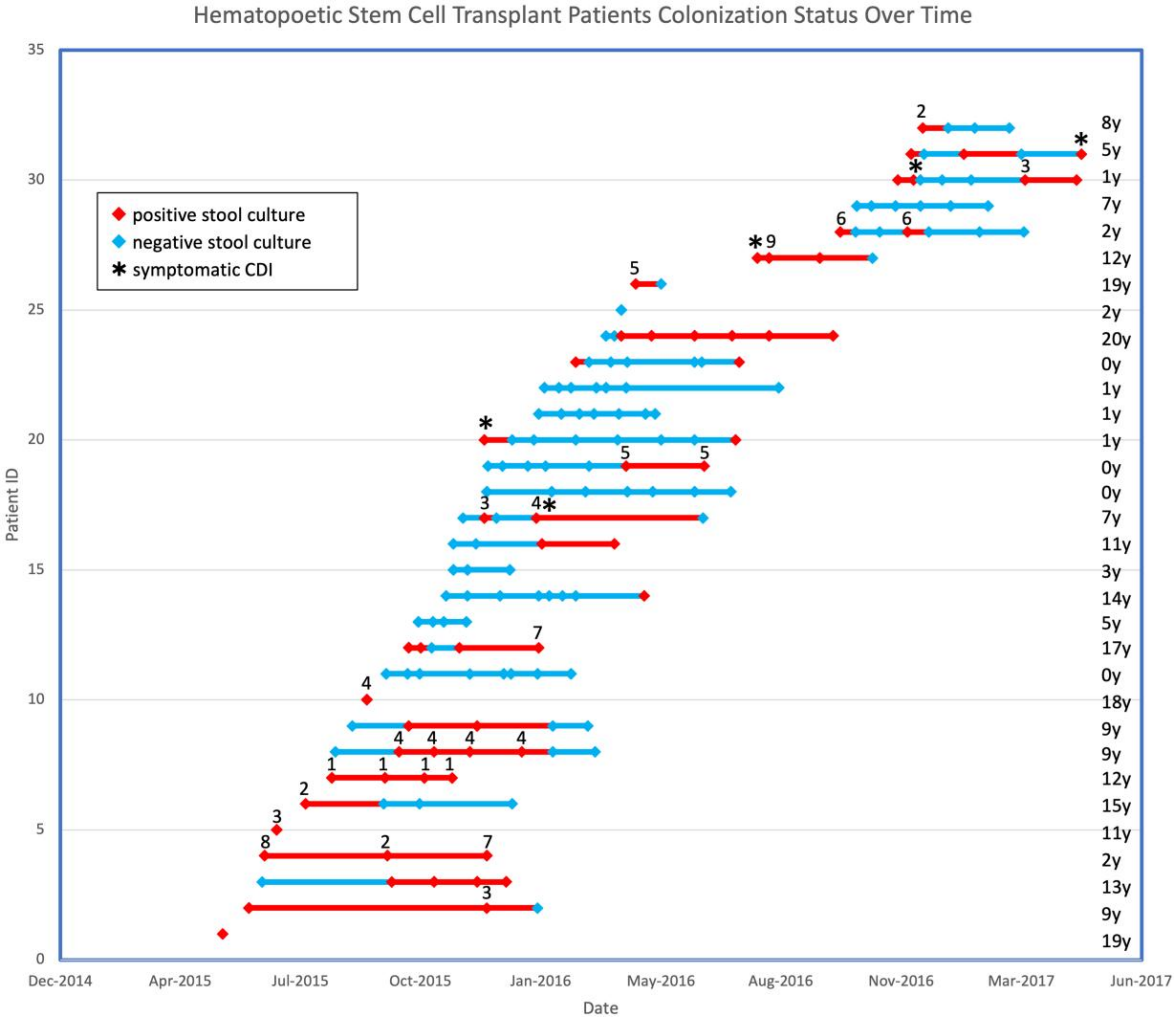
Hematopoietic stem cell transplant patients’ colonization status over time. Stools positive on culture for *Clostridioides difficile* are denoted in red, with stools negative in blue. Clonal strains found in multiple specimens were arbitrarily numbered to demonstrate persistence of strains and those found in multiple patients. Consistent numbering was used across both cohorts. *Patient with clinical diagnosis of symptomatic *C difficile* infection (CDI) at the time of stool culture.

**Figure 2.**
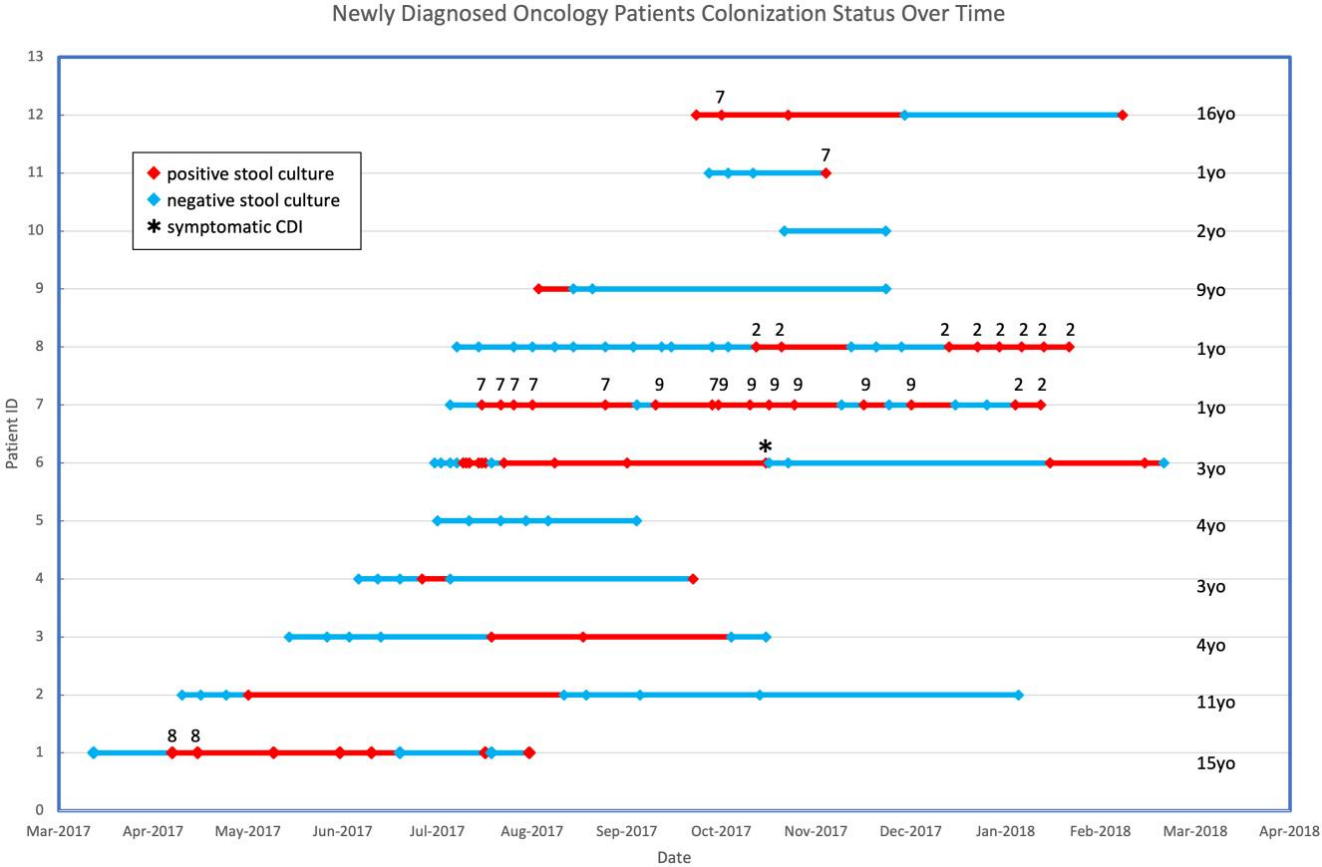
Newly diagnosed oncology patients’ colonization status over time. Stools positive on culture for *Clostridioides difficile* are denoted in red, with stools negative in blue. Clonal strains found in multiple specimens were arbitrarily numbered to demonstrate persistence of strains and those found in multiple patients. Consistent numbering was used across both cohorts. *Patient with clinical diagnosis of symptomatic *C difficile* infection (CDI) at the time of stool culture.

Of the 32 HSCT patients, 16 (50%) were colonized with *C difficile* before their transplant, including 13 (65%) of the 20 with malignancies and 3 (25%) of the 12 with nonmalignant indications for HSCT. Colonization rates rose in this population, with 75% colonized at some point in the 6 months following HSCT (Figure 1). Six (19%) HSCT patients developed clinical CDI. Only 2 of 12 (17%) newly diagnosed oncology patients were colonized with *C difficile* at baseline. However, 8 (80%) of the 10 oncology patients who were not initially colonized by *C difficile* became colonized during their cancer treatment, resulting in 83% (10/12) colonized at some point in their treatment course (Figure 2). Of HSCT patients initially negative for *C difficile*, the median (IQR) time to colonization was 63.5 days (32.5–111.5). The average time to colonization did not differ for malignant and nonmalignant transplant indications. Time to colonization was 23 days (15–50.5) for newly diagnosed oncology patients. Of the total 44 patients in both cohorts, only 10 (22.7%) remained negative for the duration of the study.

**Figure 3.**
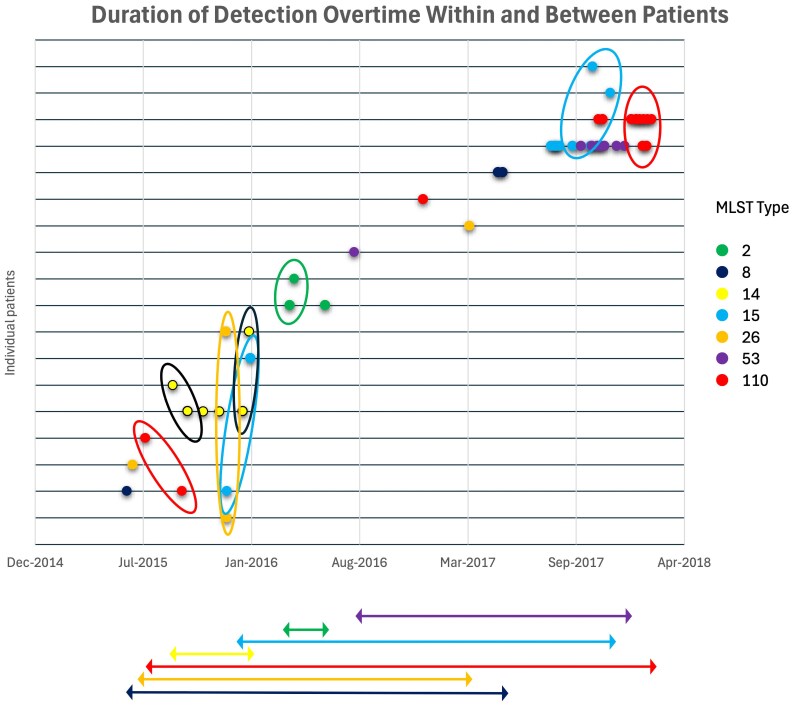
Duration and detection over time within and between patients. Individual patients are represented on the y-axis, with MLST types found in multiple patients coded by color. Ovals depict strains found in multiple patients within 2 months, suggestive of horizontal transmission. Horizonal lines demonstrate persistence of detection of clonal strains throughout the study. MLST, multilocus sequence typing.

Pairwise comparison of WGS was used to generate an SNP distance-based phylogenetic tree (Supplementary Figure 1). WGS revealed 25 MLST types. Among these, 11 were found in >1 patient. MLST 15 and 110 were each found in 5 patients. Nine clonal strains were identified in which isolates differed by <2 core MLST SNPs. The 9 strains were arbitrarily numbered for presentation purposes. Of 9 clonal strains, 7 occurred in multiple patients, with 2 clonal strains (strains 2 and 7) in 5 patients at different time points. Stools from 9 patients contained >1 strain over time. Four clonal strains were identified in both cohorts. Multiple strains persisted in our cohort over nearly 3 years. We also identified 8 time points in which clonal strains were found in multiple patients within 2 months of each other, suggesting horizontal transmission (Figure 3).

Specimens in which *C difficile* was detected by culture had greater microbiome diversity than specimens that were negative for *C difficile* by Simpson index (0.16; 95% CI, .08–.24; *P* < .001) and Shannon index (0.39; 95% CI, .11–.67; *P* = .007) for HSCT and by Simpson index in oncology patients (0.07; 95% CI, .01–.14; *P* = .03; Table 2).

**Table 2.** Microbiome Diversity in Samples Contributed by Pediatric HSCT Patients and Oncology Patients: *Clostridioides difficile* Colonized vs Uncolonized

	Differences of Least Squares Means				
Pediatric Patients: Diversity Index ^a^	Estimate	SE	*P* Value ^b^	95% CI: Lower	95% CI: Upper
HSCT					
Shannon	0.3909	0.1425	.**007**	.1089	.6729
Simpson	0.1564	0.04132	.**0002**	.07466	.2382
Inverse Simpson	0.01517	0.7284	.9834	−1.4267	1.4570
Chao	−1.5790	5.9919	.7927	−13.4743	10.3164
Oncology					
Shannon	0.1932	0.1110	.0849	−.02711	.4135
Simpson	0.07429	0.03398	.**0312**	.006848	.1417
Inverse Simpson	0.1579	0.7671	.8374	−1.3646	1.6804
Chao	−6.3008	5.4211	.2487	−17.0933	4.4918

Abbreviation: HSCT, hematopoietic stem cell transplant.

^a^The effect for each diversity index is *colonized*.

^b^Bold indicates *P* < .05.

Bacterial family abundance varied by patient cohort and by *C difficile* colonization status. The 12 most prevalent families for each are displayed in Supplementary Figure 2. Among HSCT patients, colonization was positively associated with an increased prevalence of Erysipelotrichaceae and Peptostreptococcaceae and negatively associated with Micrococcacceae, Pseudomonadacceae, Prevotellaceae, and Staphylococcaceae. Among oncology patients, colonization was associated with an increased prevalence of Peptostreptococcaceae and negatively associated with the Streptococcaceae family (Supplementary Tables 2 and 3).

## DISCUSSION

To our knowledge, this is the first longitudinal study to evaluate *C difficile* colonization in pediatric oncology patients and adds to the still-sparse literature on *C difficile* colonization in children who are immunocompromised [2, 6, 22]. By evaluating multiple stools from various time points throughout therapy, we were able to assess the duration and consistency of shedding and the acquisition of new strains. Because we used methods that reliably recover *C difficile* with as few as 10 colony-forming units per gram of stool and confirmed strain identification by matrix-assisted laser desorption ionization and WGS, we are confident that colonization was detected with high sensitivity and specificity [15]. By analyzing the *C difficile* genome, we were able to compare the likeness of isolates, and by reporting MLST, study results can be compared with longitudinal studies at other institutions.

We found that *C difficile* colonization in pediatric oncology and HSCT patients was significantly higher than previously reported in the general pediatric population and increased over the course of the patient's therapy [2]. We also demonstrated that newly diagnosed oncology patients had a low prevalence of colonization prior to receiving chemotherapy, at frequencies consistent with those expected in healthy children. While many risk factors are universally observed with CDIs in pediatric oncology patients (antibiotics, microbiome disruptions with chemotherapy, and increased hospitalizations), institutional factors might have contributed. Like many hospitals, all pediatric oncology and HSCT patients are admitted to the same inpatient unit. While valuable for specialized care, this organizational structure provides greater opportunity for interpatient transmission as staff care for colonized and uncolonized patients concurrently. Our outpatient clinic is located on the same floor as the inpatient unit, which may play an additional role in the introduction of community strains of *C difficile* to the inpatient unit.

The rate of acquisition of *C difficile* was very high. Chemotherapy and stem cell transplantation are highly disruptive to the microbiome, and it is not surprising that these treatments promote colonization. Due to the longitudinal nature of our sampling, we are able to demonstrate that the time from presentation to colonization was short, on average <2 months. As colonization can occur at any time, clinicians should be mindful that it could result in a positive test finding and falsely be interpreted as infection. For that reason, outside of research studies, testing in the absence of symptoms should be avoided. Further research is needed to explore strategies to support the microbiome and discourage *C difficile* colonization.

By performing WGS and MLST, we identified many of the same strains within the sample population. Many clonal strains were seen in patients who did not develop CDI, suggesting that silent transmission contributed to bacterial spread. While little detail is known about the movement of patients on the care units, staff interaction, environmental contamination, and direct patient contact are each potential sources for spread. For example, strain 4 was isolated from the stools of 3 patients and resulted in symptomatic CDI in 1 of them. Interestingly, Thänert et al demonstrated center-specific sharing of *C difficile* strains in unrelated preterm infants [23]. Future studies should include analysis of patient movement, which may support these findings. Moreover, these findings support increased emphasis and adherence to contact precautions and isolation efforts for patients with CDI. MLST also allows for local comparison. Many MLST types in our cohort have been described in local populations, but only 1 (MLST 11) was associated with severe disease [24]. Furthermore, through WGS, we were able to demonstrate that many strains were toxigenic but not all (Supplementary Figure 1). Colonization with nontoxigenic strains may increase risk of positive *C difficile* test results while not increasing risk for CDI.

We demonstrated a relationship between *C difficile* colonization and CDI. Seven patients in this study were diagnosed with CDI, and 3 were colonized with the same MLST type prior to symptomatic infection. In the HSCT cohort, patient 12 had MLST 42 detected 10 days before symptomatic CDI, and patient 30 had MLST 37 isolated 13 days prior to CDI. Similarly, in the oncology cohort, MLST 190 was detected 45 days before symptomatic CDI in patient 6. As CDI can disrupt and delay chemotherapy, this study provides important data to help guide potential prevention measures [4]. Additional studies are needed to investigate the relationship of colonization and infection of a strain within a host.

Patients were found to undergo *C difficile* strain turnover, with 2 patients demonstrating colonization with 3 distinct *C difficile* strains at different time points, suggesting clearance and recolonization. While it is possible that sampling error may have contributed to this finding, the conclusion is supported by the WGS. Clonal strains were most often found in successive samples, followed by ≥1 negative cultures, in turn followed by detection of a new strain. This suggests that *C difficile* colonization is a dynamic process and that further efforts to understand this process may guide interventions to alter colonization and thus infection. While colonization remains the focus of this article, clearance of colonization was seen in 23 patients. Strong conclusions cannot be made about this, as sampling error may lead to false negatives; however, our data show multiple examples of patients with several sequential negative cultures. Future studies should evaluate the factors related to medication exposure, microbiome, and the patient that promote decreased shedding of CDI and clearance of colonization.

Surprisingly, we found colonization to be associated with increased diversity, in contrast to studies reporting that CDI is associated with decreased diversity [11, 25, 26]. It is unclear why we observed this discrepant finding, although microbiome composition may affect the risk for colonization and CDI differently [27]. One hypothesis is that commensal bacteria may be present, preventing progression to CDI without inhibiting colonization [27]. Many oncology and HSCT patients also receive broad-spectrum antibiotics, and it is possible that patients received agents with activity against *C difficile*, thereby affecting biodiversity and colonization [28]. Unfortunately, these variables were not available for analysis. Further studies should investigate the modifiable factors, including medication exposure and the microbiome, which influence *C difficile* acquisition and, similarly, the factors associated with clearance of colonization. Given the high rate of colonization and the brief period prior to acquisition, this population lends itself to this inquiry.

We acknowledge several study limitations. This secondary analysis based on deidentified data was institutional review board exempt, so we had limited clinical data to fully evaluate risk factors for colonization. We therefore lacked additional variables and information in the evaluation of transmission or contamination, including co-admission, bed assignments, and staff assignments. This limitation also affects the microbiome analysis, as medication exposures in particular can greatly affect the microbiome. If available, these data would have strengthened our ability to draw conclusions. The number of samples varied, as did the interval in between sampling times for each patient. As we have no knowledge of colonization or stool microbiome data in the absence of samples, we are unable to conclusively determine the exact time when colonization occurs or clears or the exact duration of that status. Further studies could examine patient movement, bed assignments, and staff interactions to better characterize the opportunity for transmission. Sample size is a further limitation. The oncology cohort consisted of only 12 patients, which limits the generalizability of the findings. All available specimens were utilized, and as each patient contributed multiple stools, we were able to perform analysis with repeated measures, although a larger cohort may provide deeper insight. Stools were frozen from previous studies, but the aliquots studied were not subjected to freeze-thaw cycles. Nevertheless, previous studies have documented the ability to culture *C difficile* in stools frozen up to 2 years, so we believe that this had minimal impact on our ability to detect colonization [29]. WGS was also performed on only 1 or 2 colonies collected from the agar plate. If there were multiple strains growing on the plate, there is a chance that we had a sampling error that would miss the presence of multiple strains. Yet, as strains consistently clustered in time within patients, the possibility of sampling error is low.

In conclusion, *C difficile* colonization in children receiving cancer therapy or children undergoing HSCT was common in our study. Colonization was dynamic with changes in strains colonized within a host and suggestion of frequent asymptomatic transmission between patients. We were able to document instances of colonization before symptomatic CDI, which may represent an important window for infection prevention. Further investigations could assess the patient, treatment, and environmental factors that promote colonization as well as clearance of colonization. Validation studies should investigate the importance of *C difficile* environmental contamination and patient transmission as contributing factors to the high prevalence of *C difficile* colonization and explore the influence of colonization on subsequent CDI.

## Supplementary Material

ofag149_Supplementary_Data

## Supplementary Data

Supplementary materials are available at *Open Forum Infectious Diseases* online. Consisting of data provided by the authors to benefit the reader, the posted materials are not copyedited and are the sole responsibility of the authors, so questions or comments should be addressed to the corresponding author.
