# Supplementary material for: Clostridioides difficile Colonization and Infection in Pediatric Oncology and Stem Cell Transplant Patients
Source: Open Forum Infect Dis. 2026 Mar 21;13(4):ofag149. doi: 10.1093/ofid/ofag149 (PMC13037757; doi:10.1093/ofid/ofag149)
Supplement: ofag149_Supplementary_Data [file ofag149_supplementary_data.docx]

**Suppl Table 1. Patient Characteristics of Colonized Patients Stratified by timing of Colonization**

|  | Colonized at first sampling | Non-colonized at first sampling |
| --- | --- | --- |
| Cohort | 18 | 26 |
| HSCT | 16 | 16 |
| Oncology | 2 | 10 |
| Age in years at initial sample, median (IQR) | 10 (2.75-15.75) | 3.5 (1-9) |
| MLSTs | 2, 3, 8, 10, 11, 13, 14, 15, 17, 21, 26, 37, 42,  49, 53, 110, 125 | 2, 3, 8, 14, 15, 26, 28, 37, 39, 43, 53, 109, 110, 190, 205, 223, 254 |
| Time, in days, from first specimen to colonization, median (IQR) |  | 42 (19.5-79.25) |
| HSCT |  | 63.5 (39.75-109.75) |
| Oncology |  | 23 (17.5-43.75) |

Suppl Figure 1: Taxonomic tree of *C. difficile* isolates with MLST typing and toxin production

Suppl Figure 2: Abundance of Bacterial Family Stratified by Cohort and Colonization Status


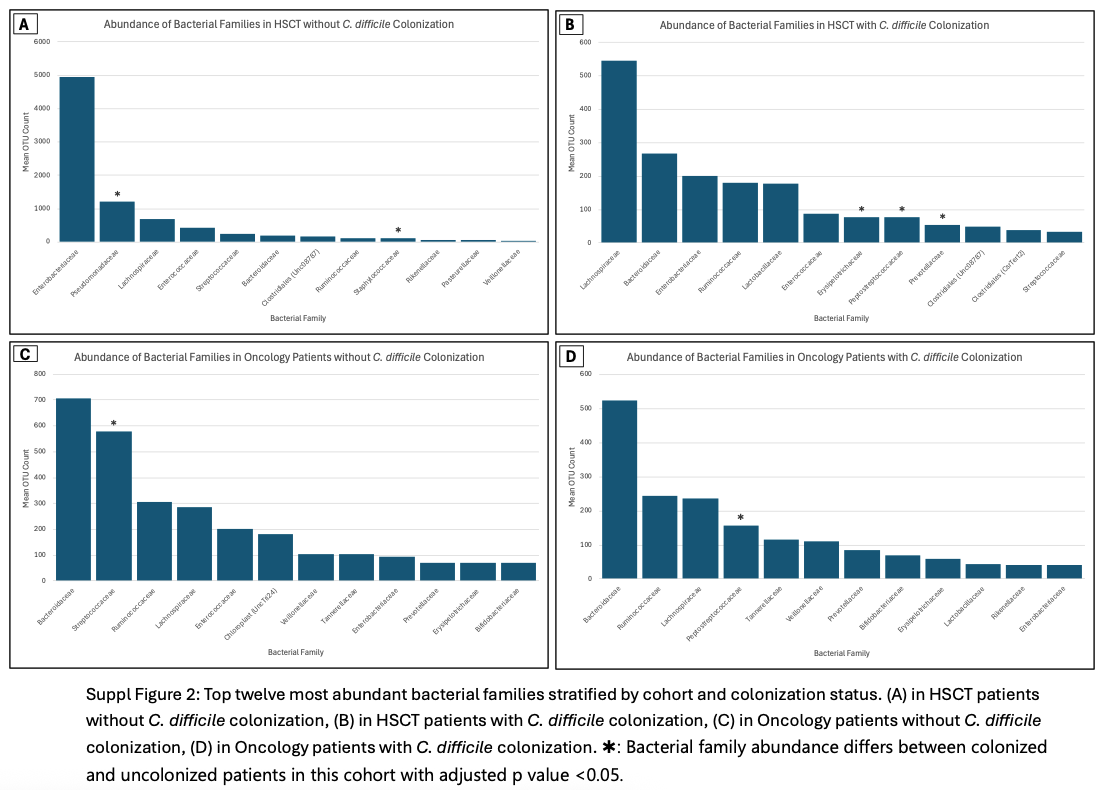


Suppl Table 2: Bacterial Composition and Comparison in Uncolonized vs Colonized Stools in the HSCT Cohort

| Bacterial Family | OTU in Uncolonized stools | OTU in Colonized stools | baseMean | log2FoldChange | lfcSE | stat | pvalue | padj | log2FoldChange_Shrink | lfcSE_Shrink |
| --- | --- | --- | --- | --- | --- | --- | --- | --- | --- | --- |
| Micrococcaceae | 22.9848 | 0.2354 | 14.9393 | -4.3774 | 0.9195 | -4.7608 | 0.0000 | 0.0001 | -2.0893 | 2.2521 |
| Pseudomonadaceae | 1199.9475 | 0.0102 | 775.5794 | -17.2182 | 4.0257 | -4.2771 | 0.0000 | 0.0006 | -0.0873 | 1.0035 |
| Erysipelotrichaceae | 24.1244 | 78.0358 | 43.1907 | 2.2763 | 0.6307 | 3.6092 | 0.0003 | 0.0063 | 1.9339 | 0.7785 |
| Peptostreptococcaceae | 15.3046 | 77.7185 | 37.3778 | 2.2505 | 0.6390 | 3.5220 | 0.0004 | 0.0066 | 1.8754 | 0.8181 |
| Prevotellaceae | 30.3319 | 54.0014 | 38.7028 | -4.7894 | 1.4723 | -3.2530 | 0.0011 | 0.0142 | -0.5471 | 1.1405 |
| Staphylococcaceae | 107.3866 | 3.8605 | 70.7737 | -4.4839 | 1.4645 | -3.0618 | 0.0022 | 0.0227 | -0.3833 | 1.0620 |
| Clostridiales (Unc08787) | 169.6794 | 48.6516 | 126.8769 | 2.7812 | 1.2283 | 2.2643 | 0.0236 | 0.2086 | 0.3307 | 0.9980 |
| Lactobacillaceae | 10.1074 | 177.4885 | 69.3032 | 1.3948 | 0.7754 | 1.7987 | 0.0721 | 0.5071 | 0.7736 | 0.8520 |
| Tannerellaceae | 34.6198 | 20.1198 | 29.4917 | -1.7448 | 0.9753 | -1.7891 | 0.0736 | 0.5071 | -0.4504 | 0.9070 |
| Rikenellaceae | 72.7950 | 19.2955 | 53.8745 | 1.7290 | 1.0043 | 1.7216 | 0.0851 | 0.5279 | 0.3404 | 0.9415 |
| Enterococcaceae | 437.4481 | 86.7399 | 313.4171 | -1.3628 | 0.8477 | -1.6077 | 0.1079 | 0.6082 | -0.5480 | 0.9566 |
| Enterobacteriaceae | 4955.5797 | 199.7318 | 3273.6335 | -1.3588 | 0.8752 | -1.5525 | 0.1205 | 0.6134 | -0.4868 | 0.9301 |
| Veillonellaceae | 34.8762 | 26.4074 | 31.8811 | -1.0073 | 0.6629 | -1.5196 | 0.1286 | 0.6134 | -0.8277 | 0.7248 |
| Burkholderiaceae | 25.0478 | 5.1333 | 18.0049 | -1.3719 | 0.9546 | -1.4371 | 0.1507 | 0.6674 | -0.3604 | 0.9111 |
| Bacteroidaceae | 194.6242 | 267.8644 | 220.5262 | 0.7202 | 0.6673 | 1.0793 | 0.2804 | 0.9985 | 0.4607 | 0.7105 |
| Ruminococcaceae | 112.4926 | 179.1012 | 136.0493 | 0.7094 | 0.6640 | 1.0684 | 0.2854 | 0.9985 | 0.4597 | 0.6884 |
| Eggerthellaceae | 0.4104 | 1.8777 | 0.9293 | 0.9857 | 0.9408 | 1.0477 | 0.2948 | 0.9985 | 1.1539 | 1.1532 |
| Actinomycetaceae | 2.0997 | 0.6790 | 1.5973 | -0.9238 | 0.9136 | -1.0112 | 0.3119 | 0.9985 | -0.3777 | 0.8667 |
| Lachnospiraceae | 680.6693 | 544.3237 | 632.4495 | 0.4350 | 0.5247 | 0.8292 | 0.4070 | 0.9985 | 0.3365 | 0.4956 |
| Pasteurellaceae | 57.8908 | 2.0796 | 38.1527 | -1.2893 | 2.0898 | -0.6169 | 0.5373 | 0.9985 | -0.2137 | 1.0173 |
| Streptococcaceae | 234.0298 | 34.4686 | 163.4533 | 0.3365 | 0.5706 | 0.5898 | 0.5553 | 0.9985 | 0.2088 | 0.5620 |
| Desulfovibrionaceae | 6.4113 | 3.6512 | 5.4352 | -0.8203 | 1.7658 | -0.4646 | 0.6422 | 0.9985 | 0.1726 | 0.9893 |
| Akkermansiaceae | 25.3564 | 19.6465 | 23.3370 | 0.8586 | 1.8736 | 0.4583 | 0.6467 | 0.9985 | 0.0233 | 0.9717 |
| Coriobacteriaceae | 1.0619 | 0.4254 | 0.8368 | -1.5897 | 4.0229 | -0.3952 | 0.6927 | 0.9985 | -0.0705 | 0.9988 |
| Campylobacteraceae | 2.7113 | 0.2547 | 1.8425 | -1.0160 | 2.6894 | -0.3778 | 0.7056 | 0.9985 | -0.1087 | 0.9995 |
| Fusobacteriaceae | 24.6588 | 0.3288 | 16.0543 | 1.2944 | 4.0241 | 0.3217 | 0.7477 | 0.9985 | -0.0131 | 0.9926 |
| Neisseriaceae | 1.1274 | 0.0418 | 0.7435 | -1.1718 | 4.0236 | -0.2912 | 0.7709 | 0.9985 | -0.0953 | 1.0032 |
| Acidaminococcaceae | 3.6735 | 5.4914 | 4.3164 | -0.5572 | 2.2191 | -0.2511 | 0.8017 | 0.9985 | 0.2401 | 1.0051 |
| Clostridiales (Unc05irm) | 0.2336 | 0.0657 | 0.1743 | -1.0105 | 4.0268 | -0.2509 | 0.8019 | 0.9985 | -0.0599 | 0.9990 |
| Atopobiaceae | 0.1905 | 0.0346 | 0.1354 | -0.9363 | 4.0262 | -0.2326 | 0.8161 | 0.9985 | -0.0556 | 0.9958 |
| Clostridiales (Unc56603) | 2.6705 | 0.9374 | 2.0575 | -0.9128 | 4.0253 | -0.2268 | 0.8206 | 0.9985 | -0.0267 | 0.9992 |
| Marinifilaceae | 0.3552 | 1.7008 | 0.8311 | -0.7788 | 4.0234 | -0.1936 | 0.8465 | 0.9985 | 0.1160 | 0.9970 |
| Clostridiales (Sarcina) | 0.5817 | 0.6244 | 0.5968 | -0.7461 | 4.0271 | -0.1853 | 0.8530 | 0.9985 | -0.0225 | 0.9991 |
| Christensenellaceae | 2.9241 | 2.3898 | 2.7351 | -0.6014 | 3.3090 | -0.1817 | 0.8558 | 0.9985 | 0.0394 | 0.9951 |
| Barnesiellaceae | 3.4869 | 2.5648 | 3.1608 | -0.6480 | 3.8387 | -0.1688 | 0.8659 | 0.9985 | -0.0167 | 0.9897 |
| Eubacteriaceae | 0.8678 | 1.6044 | 1.1283 | -0.3438 | 2.0923 | -0.1643 | 0.8695 | 0.9985 | 0.1507 | 0.9765 |
| Clostridiales (Unc038bu) | 0.0716 | 0.0185 | 0.0528 | -0.6535 | 4.0273 | -0.1623 | 0.8711 | 0.9985 | -0.0291 | 1.0007 |
| Methanobacteriaceae | 1.1361 | 1.2755 | 1.1854 | -0.6282 | 4.0262 | -0.1560 | 0.8760 | 0.9985 | 0.0123 | 0.9987 |
| Clostridiales (Unc079kw) | 0.0406 | 0.0308 | 0.0371 | -0.6036 | 4.0272 | -0.1499 | 0.8809 | 0.9985 | -0.0078 | 1.0016 |
| Leptotrichiaceae | 0.0287 | 0.0038 | 0.0199 | -0.5945 | 4.0276 | -0.1476 | 0.8827 | 0.9985 | -0.0224 | 1.0001 |
| Clostridiales (Unc0894l) | 0.0795 | 0.0904 | 0.0834 | -0.5776 | 4.0274 | -0.1434 | 0.8860 | 0.9985 | -0.0035 | 1.0013 |
| Porphyromonadaceae | 0.0225 | 0.0170 | 0.0205 | -0.5770 | 4.0277 | -0.1433 | 0.8861 | 0.9985 | -0.0167 | 1.0005 |
| Clostridiales (Unc031wu) | 0.0908 | 0.4166 | 0.2060 | -0.5546 | 4.0258 | -0.1378 | 0.8904 | 0.9985 | 0.0228 | 0.9990 |
| Muribaculaceae | 2.1094 | 0.7441 | 1.6266 | -0.4042 | 2.9665 | -0.1362 | 0.8916 | 0.9985 | -0.0174 | 0.9912 |
| Clostridiales (Unc05mei) | 0.0453 | 0.0000 | 0.0293 | -0.5421 | 4.0277 | -0.1346 | 0.8929 | 0.9985 | -0.0202 | 1.0028 |
| Defluviitaleaceae | 0.0441 | 0.0185 | 0.0351 | -0.5332 | 4.0277 | -0.1324 | 0.8947 | 0.9985 | -0.0145 | 1.0010 |
| Leuconostocaceae | 0.3661 | 0.2202 | 0.3145 | -0.5225 | 4.0258 | -0.1298 | 0.8967 | 0.9985 | -0.0164 | 0.9970 |
| Bacillales (Gemella) | 1.3606 | 0.0533 | 0.8983 | -0.4242 | 3.3074 | -0.1283 | 0.8980 | 0.9985 | -0.0295 | 0.9965 |
| Chloroplast (UncTri24) | 0.0781 | 0.0630 | 0.0728 | -0.5160 | 4.0269 | -0.1281 | 0.8980 | 0.9985 | 0.0181 | 0.9999 |
| Coriobacteriales (Gt8Me197) | 0.0289 | 0.2999 | 0.1248 | -0.4750 | 4.0271 | -0.1180 | 0.9061 | 0.9985 | 0.0166 | 0.9998 |

Suppl Table 3: Bacterial Composition and Comparison in Uncolonized vs Colonized Stools in the Oncology Patient Cohort

| **Bacterial Family** | **OTU in Uncolonized stools** | **OTU in Colonized stools** | **baseMean** | **log2FoldChange** | **lfcSE** | **stat** | **pvalue** | **padj** | **log2FoldChange_Shrink** | **lfcSE_Shrink** |
| --- | --- | --- | --- | --- | --- | --- | --- | --- | --- | --- |
| Peptostreptococcaceae | 9.1914 | 156.1148 | 12.6521 | 2.5042 | 0.5338 | 4.6916 | 0.0000 | 0.0002 | 2.7409 | 0.7032 |
| Streptococcaceae | 579.5095 | 10.9671 | 37.1829 | -2.3149 | 0.5744 | -4.0302 | 0.0001 | 0.0017 | -2.2635 | 0.8815 |
| Enterococcaceae | 200.5927 | 29.4916 | 132.4196 | -2.0434 | 0.6803 | -3.0035 | 0.0027 | 0.0524 | -1.2940 | 0.9407 |
| Chloroplast (UncTri24) | 179.9288 | 0.0202 | 3.2954 | -9.9590 | 3.4037 | -2.9260 | 0.0034 | 0.0524 | -0.0122 | 0.5923 |
| Clostridiales (Peptoniphilus) | 0.0447 | 4.2659 | 1.7266 | 8.3473 | 3.4056 | 2.4511 | 0.0142 | 0.1738 | 0.0516 | 0.5946 |
| Bifidobacteriaceae | 71.3462 | 68.8038 | 70.3332 | -1.5817 | 0.6868 | -2.3031 | 0.0213 | 0.2163 | -0.9408 | 0.8712 |
| Enterobacteriaceae | 95.3001 | 40.9917 | 73.6616 | -2.2424 | 1.0509 | -2.1339 | 0.0329 | 0.2863 | -0.1803 | 0.6029 |
| Lactobacillaceae | 8.8480 | 42.9134 | 22.4209 | 1.8140 | 0.9714 | 1.8673 | 0.0619 | 0.4716 | 0.3013 | 0.6613 |
| Akkermansiaceae | 28.1590 | 22.2147 | 25.7906 | -2.6026 | 1.5576 | -1.6710 | 0.0947 | 0.6421 | -0.1590 | 0.6037 |
| Actinomycetaceae | 1.4661 | 0.5898 | 1.1169 | -1.2205 | 0.8145 | -1.4985 | 0.1340 | 0.8174 | -0.3910 | 0.6565 |
| Christensenellaceae | 11.7154 | 0.9726 | 7.4351 | -0.8659 | 0.7645 | -1.1326 | 0.2574 | 0.9917 | -0.2523 | 0.6127 |
| Pasteurellaceae | 6.8244 | 1.5341 | 4.7165 | -1.3861 | 1.3306 | -1.0417 | 0.2975 | 0.9917 | -0.0582 | 0.5826 |
| Rikenellaceae | 38.5710 | 41.5159 | 39.7443 | 0.6525 | 0.6293 | 1.0369 | 0.2998 | 0.9917 | 0.3413 | 0.5123 |
| Clostridiales (Unc08787) | 36.9290 | 40.1551 | 38.2144 | 0.8659 | 0.8723 | 0.9926 | 0.3209 | 0.9917 | 0.1755 | 0.5509 |
| Ruminococcaceae | 306.8047 | 243.0665 | 281.4090 | 0.4097 | 0.4258 | 0.9621 | 0.3360 | 0.9917 | 0.2718 | 0.3783 |
| Tannerellaceae | 102.5885 | 115.3403 | 107.6693 | -0.4755 | 0.5149 | -0.9235 | 0.3557 | 0.9917 | -0.3641 | 0.4748 |
| Burkholderiaceae | 9.2149 | 6.4487 | 7.3611 | -0.6073 | 0.6877 | -0.8832 | 0.3772 | 0.9917 | -0.6184 | 0.6777 |
| Veillonellaceae | 105.6798 | 110.6278 | 107.6513 | -0.4590 | 0.5624 | -0.8161 | 0.4145 | 0.9917 | -0.2747 | 0.4557 |
| Prevotellaceae | 72.1244 | 84.8775 | 76.8836 | 0.5540 | 0.8724 | 0.6350 | 0.5254 | 0.9917 | 0.0949 | 0.5238 |
| Coriobacteriaceae | 3.7122 | 0.5226 | 2.4413 | -0.8345 | 1.3276 | -0.6286 | 0.5296 | 0.9917 | -0.0651 | 0.5964 |
| Clostridiales (CsrTert2) | 1.7596 | 1.6835 | 1.7293 | -0.7172 | 1.1685 | -0.6138 | 0.5394 | 0.9917 | -0.1366 | 0.6007 |
| Lachnospiraceae | 287.3129 | 235.9834 | 266.8613 | 0.2708 | 0.4473 | 0.6054 | 0.5449 | 0.9917 | 0.1710 | 0.3753 |
| Bacteroidaceae | 706.3690 | 524.7599 | 634.0091 | -0.2340 | 0.3989 | -0.5867 | 0.5574 | 0.9917 | -0.1617 | 0.3371 |
| Barnesiellaceae | 2.7540 | 0.3893 | 1.8118 | -1.0356 | 1.9236 | -0.5384 | 0.5903 | 0.9917 | -0.0696 | 0.5940 |
| Acidaminococcaceae | 12.8440 | 17.5021 | 14.6999 | 0.2942 | 0.6411 | 0.4589 | 0.6463 | 0.9917 | 0.2843 | 0.5695 |
| Desulfovibrionaceae | 15.0048 | 17.9240 | 16.1679 | 0.1447 | 0.4859 | 0.2978 | 0.7658 | 0.9917 | 0.1335 | 0.4195 |
| Bacillales (Gemella) | 0.5200 | 0.2017 | 0.3931 | -0.5605 | 1.9544 | -0.2868 | 0.7743 | 0.9917 | -0.0413 | 0.5820 |
| Atopobiaceae | 0.4354 | 0.0565 | 0.2844 | -0.9508 | 3.4067 | -0.2791 | 0.7802 | 0.9917 | -0.0370 | 0.5935 |
| Marinifilaceae | 1.4036 | 0.9465 | 1.2215 | -0.3831 | 1.6099 | -0.2380 | 0.8119 | 0.9917 | -0.0381 | 0.5850 |
| Erysipelotrichaceae | 71.4753 | 59.7647 | 66.8093 | -0.1154 | 0.4907 | -0.2352 | 0.8140 | 0.9917 | -0.0645 | 0.4253 |
| Leuconostocaceae | 0.0760 | 0.0330 | 0.0589 | -0.7111 | 3.4076 | -0.2087 | 0.8347 | 0.9917 | -0.0084 | 0.5923 |
| Clostridiales (Ezakiella) | 0.3959 | 0.0402 | 0.2542 | -0.6488 | 3.4079 | -0.1904 | 0.8490 | 0.9917 | -0.0231 | 0.5911 |
| Eubacteriaceae | 0.5139 | 1.4660 | 0.8933 | 0.2275 | 1.2650 | 0.1798 | 0.8573 | 0.9917 | 0.1265 | 0.5923 |
| Campylobacteraceae | 1.1650 | 1.5876 | 1.3334 | -0.2339 | 1.4696 | -0.1591 | 0.8736 | 0.9917 | -0.0173 | 0.5757 |
| Clostridiales (Unc2li1k) | 0.0910 | 0.0000 | 0.0547 | -0.5384 | 3.4080 | -0.1580 | 0.8745 | 0.9917 | -0.0159 | 0.5928 |
| Clostridiales (Unc0894l) | 0.1777 | 0.0449 | 0.1248 | -0.5350 | 3.4080 | -0.1570 | 0.8753 | 0.9917 | -0.0156 | 0.5925 |
| Muribaculaceae | 0.1372 | 0.1121 | 0.1272 | -0.5339 | 3.4079 | -0.1567 | 0.8755 | 0.9917 | -0.0384 | 0.5926 |
| Clostridiales (Unc038bu) | 0.0647 | 0.0000 | 0.0389 | -0.4912 | 3.4081 | -0.1441 | 0.8854 | 0.9917 | -0.0125 | 0.5929 |
| Neisseriaceae | 0.0436 | 0.0000 | 0.0262 | -0.4793 | 3.4080 | -0.1406 | 0.8882 | 0.9917 | -0.0285 | 0.5931 |
| Porphyromonadaceae | 0.0400 | 0.0118 | 0.0288 | -0.4712 | 3.4081 | -0.1383 | 0.8900 | 0.9917 | -0.0135 | 0.5903 |
| Methanobacteriaceae | 3.0115 | 0.0788 | 1.8430 | -0.4564 | 3.4072 | -0.1339 | 0.8934 | 0.9917 | -0.0084 | 0.5926 |
| Clostridiales (Finegoldia) | 0.0471 | 0.0149 | 0.0343 | -0.4394 | 3.4080 | -0.1289 | 0.8974 | 0.9917 | -0.0025 | 0.5910 |
| Clostridiales (Unc018j2) | 0.1942 | 0.0000 | 0.1168 | -0.4292 | 3.4081 | -0.1259 | 0.8998 | 0.9917 | -0.0216 | 0.5928 |
| Clostridiales (Unc031wu) | 0.1306 | 0.0545 | 0.1003 | -0.4208 | 3.4079 | -0.1235 | 0.9017 | 0.9917 | -0.0131 | 0.5918 |
| Defluviitaleaceae | 0.2669 | 0.0458 | 0.1788 | -0.4135 | 3.4077 | -0.1214 | 0.9034 | 0.9917 | -0.0006 | 0.5906 |
| Aerococcaceae | 0.1381 | 0.0218 | 0.0918 | -0.3981 | 3.4080 | -0.1168 | 0.9070 | 0.9917 | -0.0174 | 0.5926 |
| Clostridiales (Unc0317w) | 0.0818 | 0.0000 | 0.0492 | -0.3849 | 3.4081 | -0.1129 | 0.9101 | 0.9917 | -0.0142 | 0.5927 |
| Micrococcaceae | 0.1377 | 0.0894 | 0.1185 | -0.3848 | 3.4078 | -0.1129 | 0.9101 | 0.9917 | -0.0096 | 0.5908 |
| Clostridiales (Unc56603) | 0.3726 | 0.0374 | 0.2390 | -0.3809 | 3.4079 | -0.1118 | 0.9110 | 0.9917 | 0.0041 | 0.5923 |
| Clostridiales (Parvimonas) | 0.1322 | 0.1678 | 0.1464 | -0.3037 | 3.4077 | -0.0891 | 0.9290 | 0.9917 | -0.0084 | 0.5910 |
